# Association of reduced glutathione levels with *Plasmodium falciparum* and *Plasmodium vivax* malaria: a systematic review and meta-analysis

**DOI:** 10.1038/s41598-023-43583-z

**Published:** 2023-09-30

**Authors:** Manas Kotepui, Kwuntida Kotepui, Aongart Mahittikorn, Hideyuki J. Majima, Jitbanjong Tangpong, Hsiu-Chuan Yen

**Affiliations:** 1 Medical Technology, School of Allied Health Sciences, Walailak University, Tha Sala, Nakhon Si Thammarat, Thailand; 2 Department of Protozoology, Faculty of Tropical Medicine, Mahidol University, Bangkok, Thailand; 3 Department of Medical Biotechnology and Laboratory Science, College of Medicine, Chang Gung University, Taoyuan, Taiwan; 4 Department of Nephrology, Chang Gung Memorial Hospital at Linkou, Taoyuan, Taiwan

**Keywords:** Malaria, Diagnostic markers

## Abstract

Reduced glutathione (GSH) is a crucial antioxidant with recognized roles in malaria pathogenesis and host response. Despite its importance, reports on the association of GSH with malaria are inconsistent. Therefore, this systematic review and meta-analysis investigated the differences in GSH levels in relation to *Plasmodium* infection. A comprehensive literature search of six electronic databases (Embase, MEDLINE, Ovid, PubMed, Scopus, and ProQuest) was conducted. Of the 2158 initially identified records, 18 met the eligibility criteria. The majority of studies reported a significant decrease in GSH levels in malaria patients compared with uninfected controls, and this was confirmed by meta-analysis (*P* < 0.01, Hedges g: − 1.47, 95% confidence interval [CI] − 2.48 to − 0.46, *I*^*2*^: 99.12%, 17 studies). Additionally, there was no significant difference in GSH levels between *Plasmodium falciparum* malaria and *P. vivax* malaria (*P* = 0.80, Hedges g:  0.11, 95% CI − 0.76 to 0.98, *I*^*2*^: 93.23%, three studies). Similarly, no significant variation was observed between symptomatic and asymptomatic malaria cases (*P* = 0.78, Hedges g: 0.06, 95% CI − 0.34 to 0.46, *I*^*2*^: 48.07%, two studies). In conclusion, although GSH levels appear to be generally lower in malaria patients, further detailed studies are necessary to fully elucidate this complex relationship.

## Introduction

Malaria is a life-threatening disease caused by protozoan parasites of the *Plasmodium* genus that are transmitted to humans through infecting bites of female *Anopheles* mosquitoes^1^. Among them, *Plasmodium falciparum* and *P. vivax* are the most prevalent and cause the most significant public health burden^2^. Malaria is characterized by cycles of fever, chills, and sweats. Severe cases can lead to complications, such as cerebral malaria, severe anemia, and multiorgan failure^3^. During the intraerythrocytic stage, *Plasmodium* parasites metabolize hemoglobin, producing heme as a by-product^4^. Oxidative stress, an imbalance between the production of reactive oxygen species (ROS) and the body’s ability to neutralize their harmful effects through antioxidants, has been implicated in malaria pathogenesis and progression^5^. To detoxify heme, the parasite polymerizes it into hemozoin. However, this process also generates free radicals, thereby inducing oxidative stress in the host^4^. Additionally, during *Plasmodium* infection, ROS are produced by activated host phagocytes, such as neutrophils^6^. Enzymatic antioxidants, such as superoxide dismutases, catalase, and glutathione peroxidases (GPxs), and nonenzymatic antioxidants, such as vitamins C and E, β-carotene, and reduced glutathione (GSH), are activated by the host’s antioxidant defense system in response to oxidant stress^7^.

GSH is a pivotal nonenzymatic antioxidant in mammalian cells^8,9^. Besides its direct antioxidant activity, it plays several distinct roles. GSH acts as a cofactor for various enzymes, including GPx, glutathione S-transferases (GSTs), and glyoxalases^8^. Specifically, GPx uses GSH to detoxify peroxides, thereby converting GSH into glutathione disulfide (GSSG). Then, with the aid of the cofactor nicotinamide adenine dinucleotide phosphate hydrogen, glutathione reductase restores GSH from GSSG^8,10^. Additionally, GSH directly scavenges free radicals, such as superoxide anions, hydroxyl radicals, and nitric oxide, neutralizing their reactivity and preventing cellular damage^11^. It is also involved in regenerating other antioxidants, notably vitamins C and E^12^, and is a substrate for GPx, which reduces peroxides, including hydrogen peroxide and lipid peroxides. This function is crucial because it prevents the formation of more reactive species, like hydroxyl radicals^13^.

Despite the known roles of GSH, the results of studies examining its relationship with malaria are inconsistent and often limited by small sample sizes. Some studies have shown reduced GSH levels in malaria patients compared with uninfected controls^14–16^, while others reported increased GSH levels^17,18^ or no difference^19,20^. A comprehensive understanding of the role of GSH in *Plasmodium* infection, including the effects of specific species, clinical outcomes, and the relationship between GSH levels and parasite density, remains elusive. Our investigation addresses this knowledge gap, setting the groundwork for future research to translate our findings into tangible clinical and public health benefits. Elevated or diminished GSH levels may be critical markers of malaria’s clinical course, enabling earlier detection of severe cases and timely intervention. Furthermore, insights into the role of GSH may inform both clinical management and preventive strategies. Thus, this systematic review and meta-analysis investigated the differences in GSH levels in relation to *Plasmodium* infection, considering various *Plasmodium* species, clinical outcomes, and the correlation of GSH levels with parasite density.

## Methods

### Protocol

The protocol for this systematic review and meta-analysis was registered with PROSPERO (CRD42023434937) and performed based on the Preferred Reporting Items for Systematic Reviews and Meta-Analyses guidelines^21^.

### Research question for the systematic review

The structure of this systematic review was based on the Population, Exposure, Comparator, Outcome framework^22^ (P: participants included in the studies, E: occurrence of malaria, C: uninfected controls, and O: GSH levels).

### Search strategy

A comprehensive literature search was conducted across six electronic databases (Embase, MEDLINE, Ovid, PubMed, Scopus, and ProQuest). The search terms included “(“reduced glutathione” OR GSH OR “gamma-L-Glutamyl-L-Cysteinylglycine” OR “gamma L Glutamyl L Cysteinylglycine” OR “gamma-L-Glu-L-Cys-Gly” OR “gamma L Glu L Cys Gly”) AND (malaria OR plasmodium OR “Remittent Fever” OR “Marsh Fever” OR “Paludism)”, with slight variations for each database (Supplementary Table S1). We also searched Google Scholar and the reference lists of selected articles. Although Google Scholar is an expansive database, it was used as a supplementary literature search tool. This decision was based on its limitations: it only supports basic Boolean operators in search strings, lacks the ability to export results in bulk as citations, and displays only the first 1000 search records, which cannot be sorted^23^. Additionally, only the first 200 search records were screened for eligibility, as previously suggested^23^. The searches were restricted to articles written in English, but there was no restriction on the publication date. The searches began from database inception to June 12, 2023.

### Study selection and eligibility criteria

The study selection process was conducted in a stepwise manner by two independent authors (M.K. and A.M.). Any disagreement between the two authors was resolved via consensus.

First, duplicate records from the various database searches were removed manually and using automated tools. Then, the remaining unique records underwent a screening process, where irrelevant studies and those without abstracts were excluded. Next, the full texts of the remaining articles were retrieved for a detailed eligibility assessment. Studies were included in the review if they were original research articles, reported GSH levels in malaria and uninfected controls, and provided sufficient quantitative data for meta-analysis, such as the mean (with standard deviation) or median (with interquartile range) GSH levels^24^. The exclusion criteria encompassed in vitro/in vivo studies, review articles, studies lacking GSH information, studies not specifying the GSH type, studies without malaria cases, conference abstracts, or studies analyzing post-treatment GSH levels.

### Data extraction and quality assessment

The following data were extracted for each eligible study: first author’s name, year of publication, study design, year in which the study was conducted, geographic location, targeted *Plasmodium* species, clinical status, data on GSH levels, method of malaria detection, and method of GSH measurement. The quality of the included studies was assessed using Joanna Briggs Institute Critical Appraisal tools, depending on the study design (cross-sectional, cohort, or case–control)^25^. The cross-sectional studies were evaluated for clarity of inclusion criteria, validity of exposure and outcome measurements, and management of confounding factors. The cohort studies were appraised based on group similarity, validity of exposure measurement, strategies for handling confounding factors, adequacy of the follow-up period, and appropriateness of statistical analyses. The case–control studies were assessed for group comparability, appropriateness of case–control matching, validity of exposure measurement, management of confounding factors, and sufficiency of the exposure period. Responses were categorized as “Yes,” “No,” “Unclear,” or “Not applicable” based on the relevance and availability of information for each criterion. The quality rank of an individual study was determined by the percentage of “Yes” responses among all items as follows: > 75th percentile, high quality; 50th–75th percentile, moderate quality; and < 50th percentile, low quality^26^.

### Data synthesis and statistical analysis

The extracted data were used for qualitative synthesis. Additionally, a meta-analysis was conducted for quantitative synthesis. For the meta-analysis, the standardized mean difference (Hedges g) of GSH levels between groups of participants was calculated along with their 95% confidence intervals (CIs). Heterogeneity was quantified using the *I*^*2*^ statistic^27^ as follows: 0–40%, low heterogeneity; 30–60%, moderate heterogeneity; 50–90%, substantial heterogeneity; and 75–100%, considerable heterogeneity^27^. To explore the potential sources of heterogeneity, a meta-regression analysis using various factors, like the publication year, study design, continent, participant groups, *Plasmodium* species, diagnostic method for malaria, and quality rank of included studies, was conducted^28^. Subgroup analysis was conducted based on publication year, study design, geographic location, participant group, *Plasmodium* species, diagnostic method for malaria, and quality rank of included studies. A sensitivity analysis was performed using the fixed-effect model and the leave-one-out meta-analysis^29^. The leave-one-out meta-analysis was used to determine the effect of each individual study on the pooled effect estimate of the remainder of the studies^29^. Publication bias was evaluated by funnel plot analysis and Egger regression test^30^. All statistical analyses were performed using Stata v17.0 software (StataCorp. College Station, TX). *P*-values < 0.05 were considered statistically significant.

## Results

### Search results

**Figure 1:**
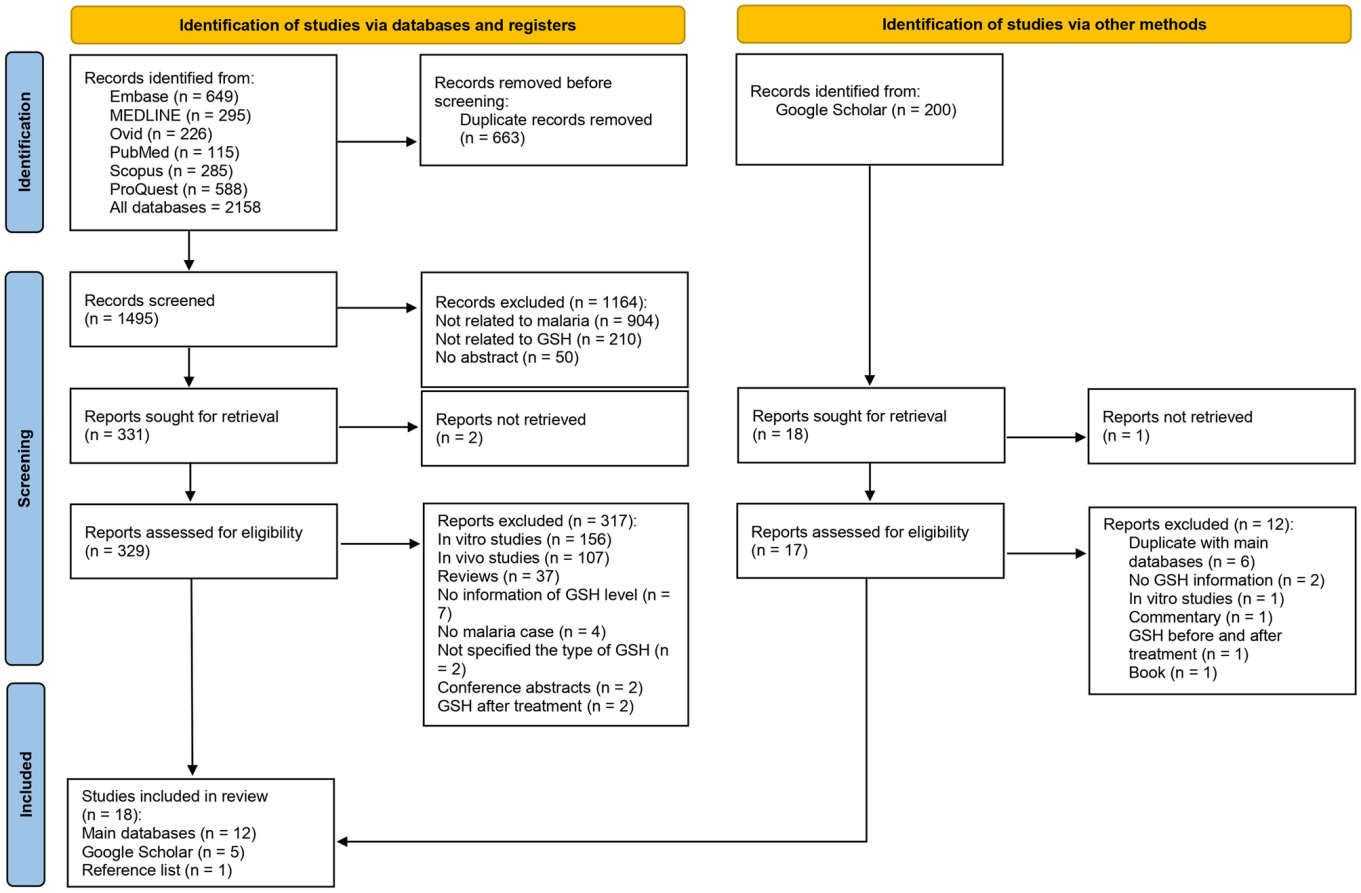
Flow diagram of the study selection process.

The searches yielded a total of 2158 records from six databases, including Embase, MEDLINE, Ovid, PubMed, Scopus, and ProQuest (n = 649, 295, 226, 115, 285, and 588 records, respectively). Initially, 663 duplicate records were eliminated, resulting in 1495 unique records for screening. Of them, 1164 were excluded due to their irrelevance to malaria or GSH or a lack of abstract, leaving 331 records for retrieval. Two records were irretrievable, so 329 reports were assessed for eligibility. This led to the exclusion of 317 records for various reasons, including being in vitro/in vivo studies, review articles, or conference abstracts, as well as lacking GSH information, the absence of malaria cases, not specifying GSH type, or analyzing post-treatment GSH levels. Eventually, 18 studies meeting the criteria were included in the review^14–20,31–41^: 12 from the primary database search^15–17,20,31–37,39^, five from Google Scholar^14,18,19,38,41^, and one from a reference list^40^ (Fig. 1).

### Characteristics of the included studies

The 18 included studies showed diverse characteristics. The majority were published during 2010–2023 (83.3%), indicating a greater interest in the investigation of the association between oxidative stress and malaria during this period. Of the studies included, only 50% specified the year in which they were conducted. The research designs were primarily cross-sectional studies (61.1%) and case–control studies (27.8%). Geographically, the studies were fairly evenly distributed between Africa (55.6%) and Asia (44.4%), with Nigeria and India being the predominant countries, respectively. *Plasmodium* species targeted in these studies were mostly *P. falciparum* (72.2%, n = 13), indicating its global importance. Although participant demographics varied, suggesting the universal impact of malaria, it is notable that approximately a quarter of the studies (27.8%) focused on children. The common method of malaria detection was light microscopy (72.2%) (Tables 1, 2, Supplementary Table S2).

**Table 1:** Characteristics of the included studies.

Characteristics	Number of studies (N = 18)	%
**Publication year**		
2010–2023	15	83.3
Before 2010	3	16.7
**Study designs**		
Cross-sectional study	11	61.1
Case–control study	5	27.8
Cohort study	2	11.1
**Study areas**		
Africa	10	55.6
Nigeria	8	44.4
Cameroon	1	5.56
Uganda	1	5.56
Asia	8	44.4
India	5	27.8
Indonesia	1	5.56
Pakistan	1	5.56
Turkey	1	5.56
***Plasmodium*** **species**		
*P. falciparum*	13	72.2
*P. falciparum*, *P. vivax*	3	16.7
*P. vivax*	2	11.1
**Participants**		
Children	5	27.8
Adults	4	22.2
All age groups	3	16.7
Not specified	4	22.2
Pregnant women	1	5.56
Pregnant and nonpregnant women	1	5.56
**Methods for malaria detection**		
Microscopy	13	72.2
Microscopy/RDT	3	16.7
RDT	1	5.56
Not specified	1	5.56

*RDT* rapid diagnostic test.

**Table 2:** Characteristics of each study.

No	Authors	Study location	Year when study was conducted	Age range	*Plasmodium* species	Clinical malaria (symptomatic or asymptomatic)	Finding regarding GSH levels	Method for malaria detection	Method for determining GSH levels
1	Abduljalil et al., 2021	Nigeria	Not specified	0–6 years	*P. falciparum*	Not specified	GSH levels were significantly decreased in malaria compared with uninfected controls	RDT	Patterson and Lazarow, 1955
2	Abubakar et al., 2016	Nigeria	2014	1–10 years	*P. falciparum*	Not specified	GSH levels were significantly decreased in malaria compared with uninfected controls	Light microscopy	Patterson and Lazarow, 1955
3	Akanbi et al., 2010	Nigeria	Not specified	Pregnant and nonpregnant women aged 16–35 years	*P. falciparum*	Not specified	GSH levels were significantly decreased in malaria compared with uninfected controls	Light microscopy	Jollow et al., 1974
4	Aqeel et al., 2019	India	Not specified	Not specified	*P. falciparum*, *P. vivax*	Symptomatic malaria	1. GSH levels were significantly decreased in *P. vivax* malaria compared with uninfected controls 2. No difference in GSH levels between *P. falciparum* malaria compared with uninfected controls 3. GSH levels were significantly decreased in *P. vivax* malaria compared with *P. falciparum* malaria	Light microscopy/RDT	Sedlak and Lindsay, 1968
5	Atiku et al., 2019	Uganda	2015–2016	Not specified	*P. falciparum*	Symptomatic malaria	1. GSH levels were significantly decreased in malaria compared with uninfected controls 2. GSH levels were similar between patients with sickle cell disease and malaria and those with sickle cell disease and no malaria (controls)	Light microscopy	Tietze et al., 1969
6	Babalola et al., 2020	Nigeria	Not specified	Not specified	*P. falciparum*	Symptomatic and asymptomatic malaria	1. No difference in GSH levels between malaria compared with uninfected controls 2. No difference in GSH levels between symptomatic and asymptomatic malaria 3. No association between GSH levels and parasite density	Light microscopy	Beutler et al., 1963
7	Bhattacharya and Swarup-Mitra, 1987	India	Not specified	8–50 years	*P. vivax*	Symptomatic malaria	1. GSH levels were significantly decreased in malaria compared with uninfected controls 2. There was a negative relationship between GSH levels and parasite density	Light microscopy	Beutler et al., 1963
8	Das and Nanda, 1999	India	1994–1995	2–12 years	*P. falciparum*	Symptomatic malaria	GSH levels were significantly decreased in malaria compared with uninfected controls	Light microscopy	Beutler et al., 1986
9	Erel et al., 1997	Turkey	Not specified	15–35 years	*P. vivax*	Symptomatic malaria	GSH levels were significantly decreased in malaria compared with uninfected controls	Light microscopy	Hebbel et al., 1986
10	Fitri et al., 2016	Indonesia	2009–2010	Not specified	*P. falciparum*	Symptomatic malaria	No difference in GSH levels between severe malaria and nonsevere malaria	Not specified	*Oxis*Research kit (catalog number 21040)
11	Javeed et al., 2011	Pakistan	Not specified	20–50 years	*P. falciparum*, *P. vivax*	Not specified	GSH levels were significantly decreased in malaria compared with uninfected controls	Light microscopy	Pulido et al., 2005
12	Nsonwu-Anyanwu et al., 2019	Nigeria	2016	18–60 years	*P. falciparum*	Not specified	GSH levels were significantly decreased in malaria compared with uninfected controls	Light microscopy	Bulaj et al., 1998
13	Ojongnkpot et al., 2023	Cameroon	2019–2021	1–15 years	*P. falciparum*	Symptomatic and asymptomatic malaria	1. GSH levels were significantly decreased in malaria compared with uninfected controls 2. There was a negative relationship between GSH levels and parasite density	Light microscopy	Ellman et al., 1959
14	Oluba, 2019	Nigeria	2015	0–5 years	*P. falciparum*	Symptomatic malaria	GSH levels were significantly decreased in both uncomplicated and severe malaria compared with uninfected controls	Light microscopy	Moron et al., 1979
15	Onyeneke et al., 2018	Nigeria	Not specified	Pregnant women	*P. falciparum*	Not defined	1. GSH levels were significantly decreased in malaria compared with uninfected controls 2. No association between GSH levels and parasite density	Light microscopy	Ellman et al., 1959
16	Ozojiofor et al., 2021	Nigeria	2018–2019	10–60 years	*P. falciparum*	Symptomatic malaria	GSH levels were significantly decreased in malaria compared with uninfected controls	Light microscopy/RDT	Jollow et al., 1974
17	Sohail et al., 2010	India	Not specified	Not specified	*P. falciparum*, *P. vivax*	Not specified	GSH levels were significantly increased in malaria compared with uninfected controls	Light microscopy/RDT	Jollow et al., 1974
18	Tyagi et al., 2017	India	2012–2013	13–82 years	*P. falciparum*	Symptomatic malaria	GSH levels were significantly increased in malaria compared with uninfected controls	Light microscopy	Beutler et al. (year unspecified)

*GSH* reduced glutathione, *RDT* rapid diagnostic test.

### Quality of the included studies

For the cross-sectional studies, the majority^15,16,20,32,35,40^ exhibited robust adherence to the predetermined criteria but failed to address confounding factors. However, three studies^17,19,38^ successfully met all quality parameters. Two studies^36,41^, although fulfilling most criteria and identifying confounding factors, neglected to proffer strategies to mitigate confounders. Regarding the cohort studies, only one^34^ met all quality parameters. However, the other cohort study^39^ displayed certain inadequacies and ambiguities, including exposure measurement and handling of confounding factors. For the case–control studies, one^14^ struggled with group comparability and case–control matching and did not address confounding factors. Another^31^ upheld all quality criteria. The remaining three case–control studies^18,33,37^, while fulfilling most criteria, were vague regarding case–control matching and the duration of the exposure period (Supplementary Table S3).

### Qualitative synthesis

The majority of African studies on GSH levels in malaria were conducted in Nigeria, with significant contributions from authors such as Abduljalil et al.^14^, Abubakar et al.^15^, Akanbi et al.^16^, Babalola et al.^19^, Nsonwu-Anyanwu et al.^37^, Oluba et al.^39^, Onyeneke et al.^40^, and Ozojiofor et al.^41^. The majority of Asian studies were conducted in India, as evidenced by works from Aqeel et al.^31^, Bhattacharya and Swarup-Mitra^33^, Das and Nanda^34^, Sohail et al.^17^, and Tyagi et al.^18^. Overall, a significant decrease in GSH levels in individuals with malaria compared with uninfected controls was observed in 13 of these studies. Regarding different age groups, the majority of studies focused on children observed a significant decrease in GSH levels when infected with malaria, as seen in studies by Abduljalil et al.^14^, Abubakar et al.^15^, Das and Nanda^34^, Oluba et al.^39^, and Ojongnkpot et al.^38^. In particular, Ojongnkpot’s study from Cameroon highlighted a negative relationship between GSH levels and parasite density in children^38^. A decline in GSH levels in malaria patients was predominantly observed in studies on adults, including studies by Erel et al. in Turkey^35^, Javeed et al. in Pakistan^36^, and Nsonwu-Anyanwu et al. in Nigeria^37^. In Indonesia, Fitri et al.^20^ noted no difference in GSH levels between severe and nonsevere malaria in adults. Two Nigerian studies specifically focused on pregnant and nonpregnant women: Akanbi et al.^16^ and Onyeneke et al.^40^. Both reported decreased GSH levels in subjects with malaria compared with uninfected controls. However, Onyeneke et al. did not find an association between GSH levels and parasite density in this group^40^. Some studies enrolled participants from all age groups, with Bhattacharya and Swarup-Mitra^33^ and Ozojiofor et al.^41^, from India and Nigeria, respectively, noting a decline in GSH levels in malaria patients. In contrast, the Indian studies by Sohail et al.^17^ and Tyagi et al.^18^ observed increased GSH levels in malaria patients, providing an intriguing counterpoint.

### GSH levels between malaria and uninfected controls

**Figure 2:**
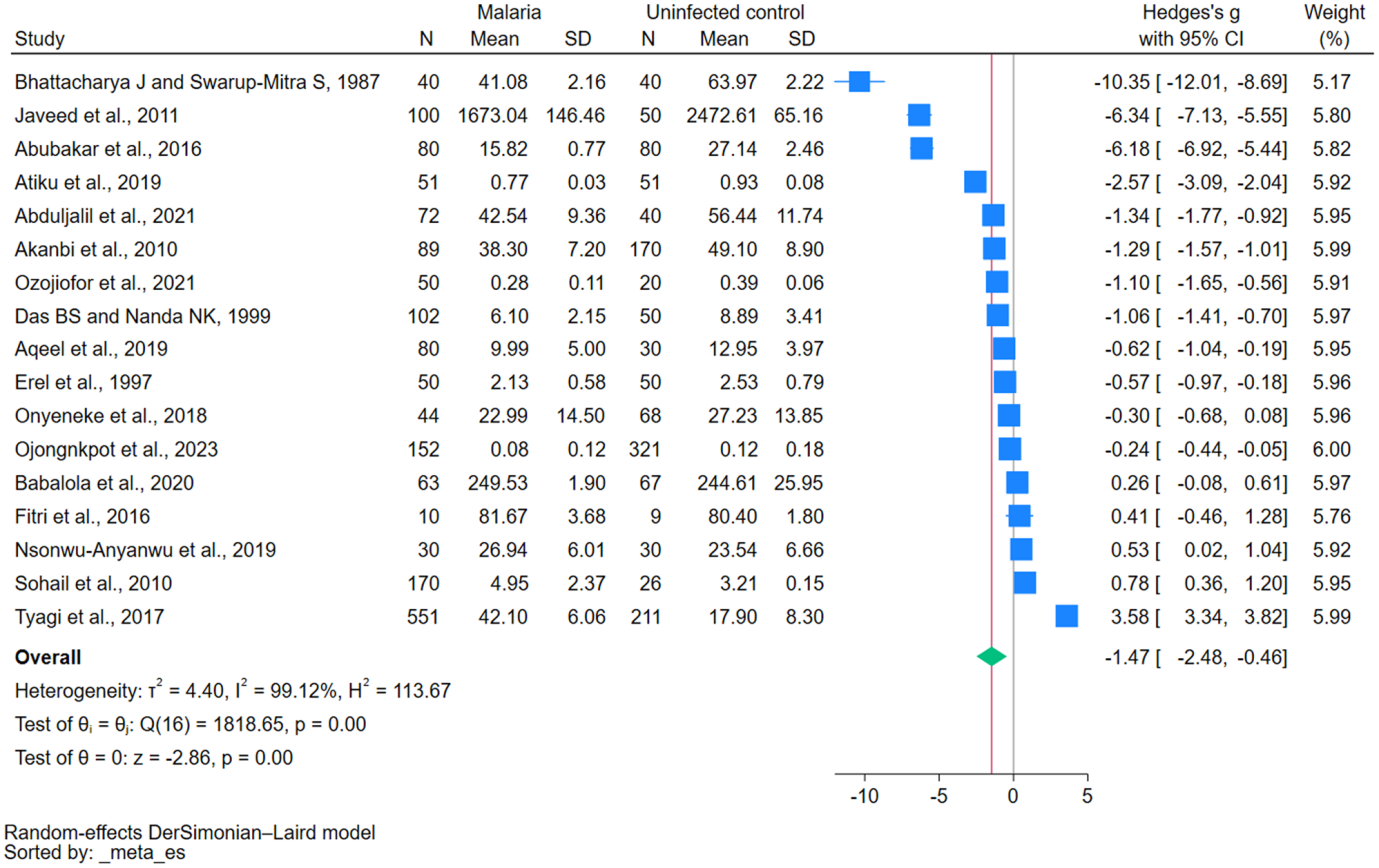
Forest plot showing the difference in the reduced glutathione levels between malaria patients and uninfected controls. *CI* confidence interval, *N* number of participants, *SD* standard deviation, *blue square* effect estimate, *green diamond* pooled effect estimate, *red vertical line* pooled effect estimate, *gray vertical line* no effect line.

A total of 17 studies investigated GSH levels in both malaria and uninfected controls and reported quantitative data, which we used in the meta-analysis^14–20,31–38,40,41^. Compared with uninfected controls, GSH levels were reduced in 11 malaria studies^14–16,31–36,38,41^, increased in three malaria studies^17,18,37^, and showed no difference in three malaria studies^19,20,40^. The meta-analysis result showed the reduction of GSH in malaria compared with uninfected controls (*P* < 0.01, Hedges g: − 1.47, 95% CI − 2.48 to − 0.46, *I*^*2*^: 99.12%, 17 studies; Fig. 2). The meta-regression analysis, which considered factors such as publication year, study design, continent, participant groups, *Plasmodium* species, diagnostic methods for malaria, and the quality rank of the included studies, showed that only the *Plasmodium* species influenced the pooled effect estimate (Supplementary Table S4). This suggests that different *Plasmodium* species may influence variations in GSH levels in patients with malaria.

The subgroup analysis results are shown in Table 3. Studies published from 2010–2023 indicated no significant difference in GSH levels between the groups (*P* = 0.08, Hedges g =  − 1.01, 95% CI: − 2.15 to 0.12), whereas studies conducted before 2010 showed a significant difference (*P* < 0.01, Hedges g =  − 3.69, 95% CI − 6.09 to − 1.29). Cross-sectional studies showed significant differences in GSH levels between malaria cases and controls (*P* < 0.01, Hedges g =  − 1.53, 95% CI − 2.44 to − 0.62), while case–control studies did not (*P* = 0.27, Hedges g =  − 1.52, 95% CI − 4.23 to 1.19). Geographically, studies from Africa demonstrated significant differences (*P* < 0.01, Hedges g =  − 1.32, 95% CI − 2.16 to − 4.09), but those conducted in Asia did not (*P* = 0.11, Hedges g =  − 1.69, 95% CI − 3.74 to 0.37). Regarding participant groups, only studies involving children showed a significant difference in GSH levels (*P* = 0.01, Hedges g =  − 2.16, 95% CI − 3.80 to − 0.53), while others did not. Differences in *Plasmodium* species (*P. falciparum*, *P. vivax*, or a mix of both species) did not show significant differences in GSH levels. In terms of diagnostic methods for malaria, studies using microscopy showed significant differences (*P* < 0.01, Hedges g =  − 1.95, 95% CI: − 3.28 to − 0.62), but those using a combination of microscopy and rapid diagnostic tests did not (*P* = 0.59, Hedges g =  − 0.30, 95% CI − 1.42 to 0.81). Concerning the studies’ quality, high-quality studies showed significant differences (*P* < 0.01, Hedges g =  − 1.56, 95% CI − 2.35 to − 0.76), but moderate-quality studies did not (*P* = 0.36, Hedges g =  − 1.33, 95% CI − 4.20 to 1.54).

**Table 3:** Subgroup analyses of GSH levels between malaria cases and uninfected controls.

Subgroup analyses	*P*-value	Hedges g (95% CI)	*I*^*2*^	Number of studies (N = 17)
Publication years				
2010–2023	0.08	− 1.01 (− 2.15 to 0.12)	99.20	14
Before 2010	< 0.01	− 3.69 (− 6.09 to − 1.29)	98.41	3
Study design				
Cross-sectional study	< 0.01	− 1.53 (− 2.44 to − 0.62)	98.28	11
Case–control study	0.27	− 1.52 (− 4.23 to 1.19)	99.48	5
Cohort study	N/A	− 1.06 (− 1.41 to − 0.7)	N/A	1
Continent				
Africa	< 0.01	− 1.32 (− 2.16 to − 4.09)	97.80	9
Asia	0.11	− 1.69 (− 3.74 to 0.37)	99.41	8
Participant groups				
Children	0.01	− 2.16 (− 3.80 to − 0.53)	98.75	4
Adults	0.25	− 1.48 (− 4.03 to 1.06)	98.65	4
All age groups	0.33	− 2.54 (− 7.59 to 2.51)	99.58	3
Pregnant and nonpregnant women	N/A	− 1.29 (− 1.57 to − 1.01)	N/A	1
Pregnant women	N/A	− 0.30 (− 0.68 to 0.08)	N/A	1
Age unspecified	0.42	− 0.53 (− 1.80 to 0.75)	97.29	4
*Plasmodium* species				
*P. falciparum*	0.20	− 0.76 (− 1.94 to 0.41)	99.21	12
*P. vivax*	0.27	− 5.43 (− 15.01 to 4.15)	99.21	2
*P. falciparum*, *P. vivax*	0.22	− 2.04 (− 5.28 to 1.20)	99.18	3
Diagnostic method for malaria				
Microscopy	< 0.01	− 1.95 (− 3.28 to − 0.62)	99.37	12
Microscopy/RDT	0.59	− 0.30 (− 1.42 to 0.81)	94.37	3
RDT	N/A	− 1.34 (− 1.77 to 0.92)	N/A	1
Not specified	N/A	0.41 (− 0.46 to 1.28)	N/A	1
Quality rank of included studies				
High	< 0.01	− 1.56 (− 2.35 to − 0.76)	98.10	12
Moderate	0.36	− 1.33 (− 4.20 to 1.54)	99.40	5

*CI* confidence interval, *N/A* not assessed, *RDT* rapid diagnostic test.

### GSH levels between *P. falciparum* and *P. vivax*

**Figure 3:**
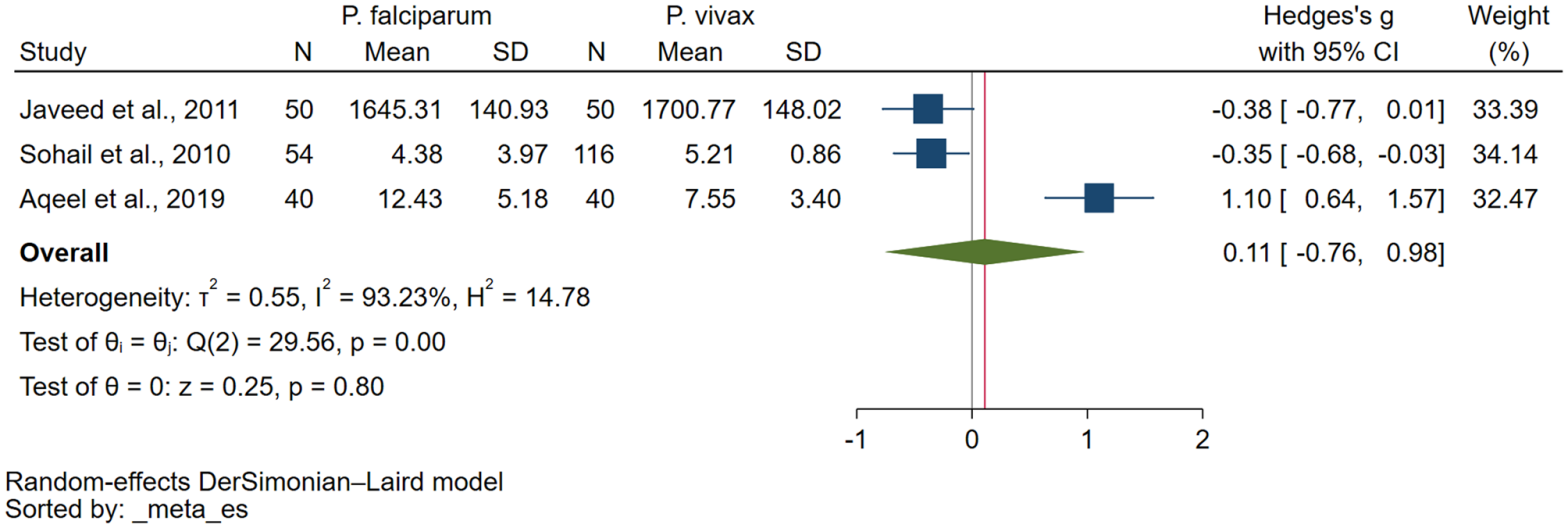
Forest plot showing the difference in the reduced glutathione levels between patients with *P. falciparum* malaria and those with *P. vivax* malaria. *CI* confidence interval, *N* number of participants, *SD* standard deviation, *blue square* effect estimate, *green diamond* pooled effect estimate, *red vertical line* pooled effect estimate, *gray vertical line* no effect line.

Three studies investigated GSH levels in both *P. falciparum* and *P. vivax* malaria cases, providing quantitative data applicable to the meta-analysis^17,31,36^. The results showed a significant difference in GSH levels between *P. falciparum* and *P. vivax* malaria (*P* = 0.80, Hedges g: 0.11, 95% CI: − 0.76 to 0.98, *I*^*2*^: 93.23%, three studies; Fig. 3). Performing both meta-regression and subgroup analysis was not feasible in this context due to the restricted number of studies available.

### GSH levels between symptomatic and asymptomatic malaria

**Figure 4:**
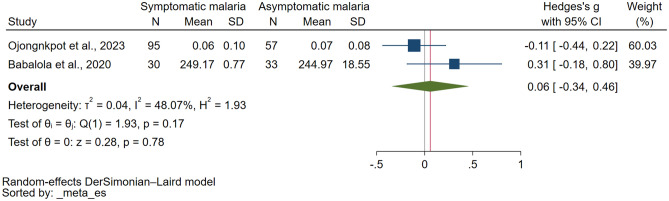
Forest plot showing the difference in the reduced glutathione levels between patients with symptomatic and asymptomatic malaria. *CI* confidence interval, *N* number of participants, *SD* standard deviation, *blue square* effect estimate, *green diamond* pooled effect estimate, *red vertical line* pooled effect estimate, *gray vertical line* no effect line.

Two studies investigated GSH levels in both symptomatic and asymptomatic *P. falciparum* malaria and reported quantitative data, which we used in the meta-analysis^19,38^. The results revealed no significant difference in GSH levels between symptomatic and asymptomatic malaria cases (*P* = 0.78, Hedges g: 0.06, 95% CI: − 0.34 to 0.46, *I*^*2*^: 48.07%, two studies; Fig. 4). Due to the limited number of studies available, both a meta-regression and subgroup analysis could not be conducted.

### Sensitivity analysis

**Figure 5:**
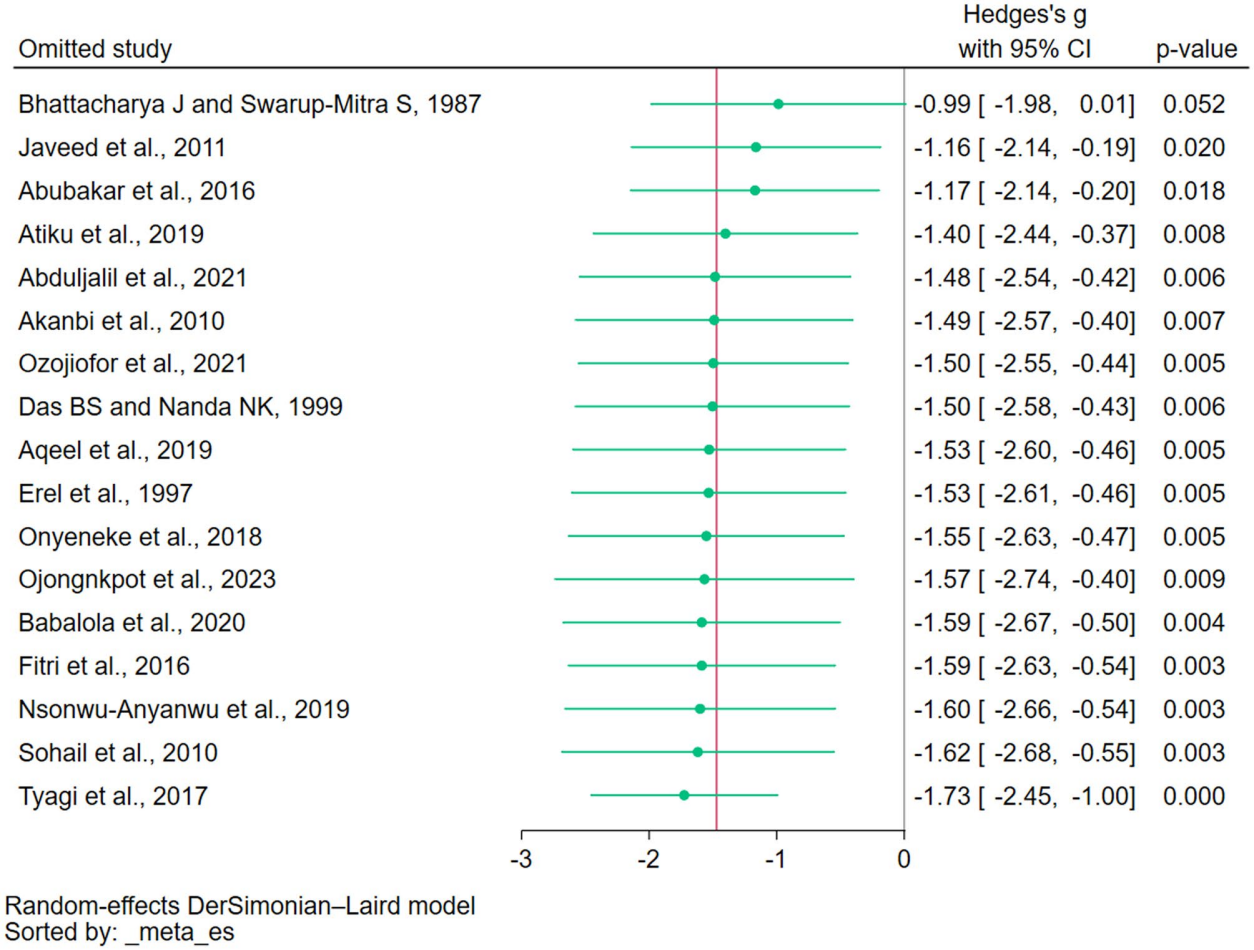
Results of the leave-one-out rerun meta-analysis of the difference in the reduced glutathione levels between malaria patients and uninfected controls. *CI* confidence interval, *green dot* pooled effect estimate, *green horizontal line* confidence interval, *red vertical line* pooled effect estimate, *gray vertical line* no effect line.

Two approaches were employed to perform sensitivity analysis: the fixed-effect model and the leave-one-out meta-analysis. The fixed-effect model revealed a significant reduction in GSH levels in individuals with malaria compared with uninfected controls (*P* = 0.01, Hedges g: − 0.12, 95% CI − 0.21 to − 0.03), as confirmed by the meta-analysis of 17 studies (*I*^*2*^: 99.12%, Supplementary Fig. S1). The leave-one-out meta-analysis pinpointed the study by Bhattacharya and Swarup-Mitra^33^ as an outlier. Its removal altered the meta-analysis results (*P* = 0.052, Hedges g: − 0.99, 95% CI − 1.98 to − 0.01; Fig. 5).

### Publication bias

**Figure 6:**
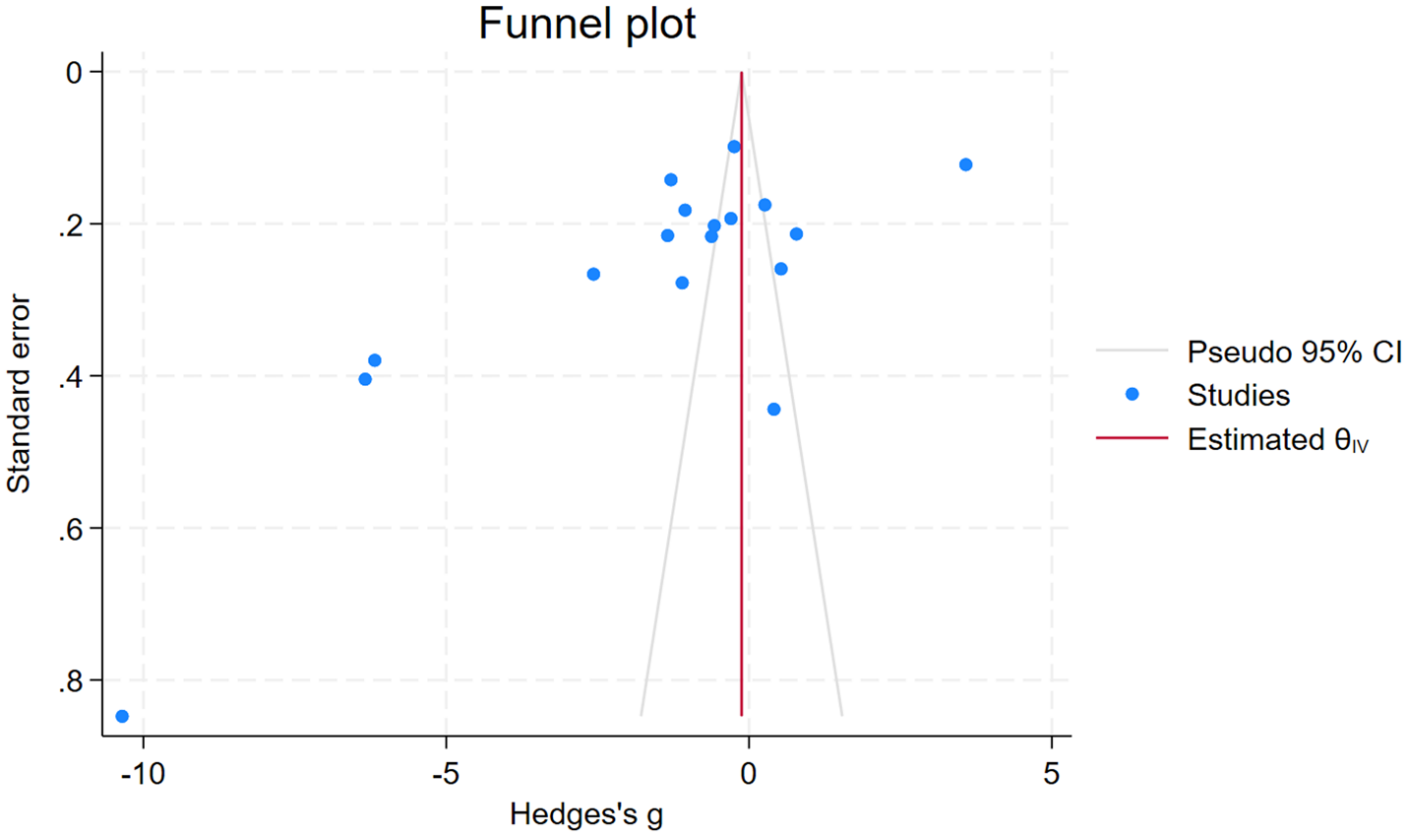
Funnel plot showing an asymmetrical distribution of the effect estimates between the middle line of the plot. *CI* confidence interval, *Blue dot* effect estimate, *red vertical line* pooled effect estimate.

Two standard methodologies were implemented to evaluate the presence of publication bias: a funnel plot analysis and Egger regression test. The results depicted in the funnel plot were asymmetrical (Fig. 6), suggesting an imbalanced distribution of studies around the mean effect size, implying substantial publication bias. Egger test was further conducted to quantify the bias captured in the funnel plot. Notably, this test identified a significant result (*P* < 0.01). Therefore, both the asymmetrical funnel plot and the significant Egger test result collectively indicate the possible presence of publication bias due to the small-study effect in the meta-analysis.

## Discussion

Qualitatively, most studies concur that GSH levels significantly decrease in malaria patients compared with uninfected controls, corroborating the role of GSH in malaria pathogenesis. Furthermore, the meta-analysis confirmed that GSH levels significantly decreased in malaria patients compared with uninfected controls. GSH, a critical antioxidant in human cells, protects the body from damage caused by oxidative stress^42^. The observed decrease in GSH levels in malaria patients may be a consequence of the body utilizing its GSH reserves to counteract the oxidative stress caused by malaria. Reductions in host GSH levels during malaria can have dual implications for disease pathogenesis. On the one hand, diminished GSH renders host erythrocytes more vulnerable to oxidative damage, potentially exacerbating disease symptoms due to increased oxidative stress^43^. Conversely, the malaria parasite, particularly *P. falciparum*, relies on host GSH to detoxify and resist antimalarial drugs^4^. Therefore, while reduced GSH may weaken the host’s defense against oxidative damage, it can also hinder the parasite’s ability to survive drug treatments. Thus, the intricate balance between host and parasite GSH dynamics underscores the complex nature of malaria pathogenesis^44^. However, this generalized trend is subject to certain exceptions. For instance, Aqeel et al. found decreased GSH levels, specifically in patients with *P. vivax* malaria but not in patients with *P. falciparum* malaria^31^. This divergence may stem from the different pathophysiological mechanisms employed by *P. falciparum* and *P. vivax*. Since *P. vivax* and *P. falciparum* exhibit different levels of disease severity, this may influence the level of oxidative stress and, consequently, GSH consumption^45^. For example, infection with *P. falciparum* can lead to more severe complications, yet some *P. vivax* cases can also develop severe malaria^46,47^. Nevertheless, no difference in GSH levels between *P. falciparum* and *P. vivax* malaria was observed, suggesting that more studies are necessary to determine differences in the distinct antioxidant levels between these two species. Furthermore, the meta-analysis of the clinical status of patients revealed no significant difference in GSH levels between symptomatic and asymptomatic malaria cases. This suggests that alterations in GSH levels are consistent, irrespective of the presence of symptoms. Additionally, a persistent reduction of plasma GSH levels in the early stages of the disease has been observed, which was later compensated during the advanced phase^31^. Thus, reduced GSH levels may be common during the acute phase of *Plasmodium* infection, regardless of disease severity or whether the infection is caused by *P. vivax* or *P. falciparum*.

Other subgroup meta-analyses demonstrated differences in GSH levels between malaria patients and controls across specific parameters, such as geographic location and participant groups. Notably, African studies showed a significant difference in GSH levels between malaria patients and controls, whereas Asian studies did not. This discrepancy may be because the African studies mainly enrolled children. In the subgroup analysis of age groups of participants, only studies involving children showed significant differences in GSH levels, possibly indicating that malaria has a more pronounced effect on GSH levels in this age group. Furthermore, the less mature immune systems of children may impact the oxidative stress response^48^. Additionally, children in Africa have lower antioxidant levels, which may lead to more severe malaria cases^49^.

Interestingly, Babalola et al.^19^ and Fitri et al.^20^ reported no significant differences in GSH levels between different patient groups. This finding may be attributed to a range of factors, including the timing of sample collection, the disease stage, and individual variations in patients’ antioxidant response. In contrast, two studies reported increased GSH levels in malaria patients^17,18^. While this may initially seem counterintuitive given the body’s typical response to oxidative stress, it is important to note that the elevated GSH levels are likely attributed to the parasite itself. In their bid to survive and resist antimalarial drugs, *Plasmodium* species can upregulate GSH synthesis, leading to observed increases in overall GSH levels within the host^50,51^. Sohail et al. proposed that the observed increase in GSH levels in malaria patients could be due to transitional polymorphisms within GSTs, which might enhance the host’s GSH availability^17^. Tyagi et al. suggested that increased GSH levels among malaria patients might be due to decreased GSH utilization^18^.

The relationship between GSH levels and parasite density varied across studies, with some reporting a negative relationship and others finding no significant association^33,38^. These discrepancies may be attributed to factors such as variations in the host’s immune response, the parasite’s lifecycle stage when the sample was collected, or differences in *Plasmodium* species. Oxidative stress and sickle cell disease are related to each other^52^. One of the included studies demonstrated that patients with malaria and sickle cell disease experienced severe oxidative stress^32^. The authors reported that although the GSH levels were higher in patients without sickle cell disease compared with those with malaria and sickle cell disease, the difference was not statistically significant. Therefore, patients with both malaria and sickle cell disease may have a higher demand for GSH to detoxify the increased oxidative stress^32^.

While the meta-analysis demonstrated a significant reduction in GSH levels in malaria patients compared with uninfected controls, indicating an association between malaria and lower levels of this critical antioxidant, the high variability among studies (*I*^*2*^: 99.12%) must not be ignored, suggesting substantial heterogeneity in the results. A limited number of studies investigated GSH levels in various contexts: (i) malaria patients with severe complications versus those without severe complications, (ii) asymptomatic versus symptomatic malaria, and (iii) *P. falciparum* versus non-*P. falciparum* malaria. Thus, the conclusions of our study were limited. Among the high-quality studies, there was a significant difference in GSH levels with a substantial effect size. Conversely, moderate-quality studies did not show a statistically significant difference. This may highlight the importance of study quality when interpreting results. Additionally, the level of heterogeneity remained high when the subgroup analyses were performed. Therefore, the true confounders of the relationship between GSH and malaria remain unidentified. Other potential confounders may include both infectious and noninfectious conditions that are co-endemic with malaria, such as nutritional deficiencies^53^, diabetes^54^, human immunodeficiency virus^55^, and coronavirus disease 2019^56^. Based on the information from the included studies, the timing of sample collection was not explicitly stated in each study. Consequently, a meta-regression analysis to test whether the timing of sample collection influenced the effect estimate of the meta-analysis could not be performed. The final limitation is the evidence of significant publication bias, and the sensitivity analysis calls for a cautious interpretation of these findings. The considerable impact of a single study on the overall meta-analysis outcome underscores the importance of incorporating a diverse range of studies to mitigate potential biases.

## Conclusion

This comprehensive review and meta-analysis of the existing literature indicate a trend of decreased GSH levels in malaria patients compared with uninfected controls, which is consistent with the majority of the reviewed studies. Furthermore, the meta-analysis underlines the potential of GSH as a diagnostic biomarker for malaria. However, the relationship between GSH levels and specific characteristics, such as *Plasmodium* species, malaria symptoms, and geographic location, revealed more nuanced findings. Further studies are necessary to corroborate these findings and delve deeper into the complex relationship between malaria and GSH levels.

### Supplementary Information

Supplementary Table S1.

Supplementary Table S2.

Supplementary Table S3.

Supplementary Table S4.

Supplementary Figure 1.

## Data Availability

All data relating to the present study are available in this manuscript and supplementary files.
