# Supplementary material for: Association of reduced glutathione levels with Plasmodium falciparum and Plasmodium vivax malaria: a systematic review and meta-analysis
Source: Sci Rep. 2023 Sep 30;13:16483. doi: 10.1038/s41598-023-43583-z (PMC10542361; doi:10.1038/s41598-023-43583-z)
Supplement: Supplementary file 1 — Supplementary Table S1. [file 41598_2023_43583_MOESM1_ESM.docx]

**Association of reduced glutathione levels with *Plasmodium falciparum* and *Plasmodium vivax* malaria: A systematic review and meta-analysis**

Manas Kotepui^1*^, Kwuntida Kotepui^1^, Aongart Mahittikorn^2*^, Hideyuki J Majima^1^, Jitbanjong Tangpong^1^, Hsiu-Chuan Yen^3^

^1^Medical Technology, School of Allied Health Sciences, Walailak University, Tha Sala, Nakhon Si Thammarat, Thailand

^2^Department of Protozoology, Faculty of Tropical Medicine, Mahidol University, Bangkok, Thailand

^3^ Department of Medical Biotechnology and Laboratory Science, College of Medicine, Chang Gung University, Taoyuan, Taiwan

^4^ Department of Nephrology, Chang Gung Memorial Hospital at Linkou, Taoyuan, Taiwan

***Corresponding author**

Manas Kotepui [manas.ko@wu.ac.th](mailto:manas.ko@wu.ac.th), Tel.: +66954392469

Kwuntida Uthaisar Kotepui: [kwuntida.ut@wu.ac.th](mailto:kwuntida.ut@wu.ac.th)

Aongart Mahittikorn: aongart.mah@mahidol.ac.th

Hideyuki J Majima: [k0941761@kadai.jp](mailto:k0941761@kadai.jp)

Jitbanjong Tangpong: rjitbanj@wu.ac.th

Hsiu-Chuan Yen: yen@mail.cgu.edu.tw

**Table S1. Search terms**

**General search strategy**

(“reduced glutathione” OR GSH OR “gamma-L-Glutamyl-L-Cysteinylglycine” OR “gamma L Glutamyl L Cysteinylglycine” OR “gamma-L-Glu-L-Cys-Gly” OR “gamma L Glu L Cys Gly”) AND (malaria OR plasmodium OR “Remittent Fever“ OR “Marsh Fever“ OR Paludism)

PubMed 12 June 2023

| No. | Key concept | Search terms | Results |
| --- | --- | --- | --- |
| 1. | Reduced glutathione | “reduced glutathione”[Text Word] OR “reduced glutathione”[Mesh] OR GSH[Text Word] OR GSH[Mesh] OR “gamma-L-Glutamyl-L-Cysteinylglycine”[Text Word] OR “gamma L Glutamyl L Cysteinylglycine”[Text Word] OR “gamma-L-Glu-L-Cys-Gly”[Text Word] OR “gamma L Glu L Cys Gly”[Text Word] | 66,886 |
| 2. | Malaria | malaria[Text Word] OR plasmodium[Text Word] OR “Infections, Plasmodium“[Mesh] OR “Infection, Plasmodium“[Mesh] OR “Plasmodium Infection“[Mesh] OR “Remittent Fever“[Mesh] OR “Fever, Remittent“[Mesh] “Marsh Fever“[Mesh] OR “Fever, Marsh“[Mesh] OR Paludism[Mesh] | 74,291 |
| 3. | 1 AND 2 | (“reduced glutathione”[Text Word] OR “reduced glutathione”[Mesh] OR GSH[Text Word] OR GSH[Mesh] OR “gamma-L-Glutamyl-L-Cysteinylglycine”[Text Word] OR “gamma L Glutamyl L Cysteinylglycine”[Text Word] OR “gamma-L-Glu-L-Cys-Gly”[Text Word] OR “gamma L Glu L Cys Gly”[Text Word]) AND (malaria[Text Word] OR plasmodium[Text Word] OR “Infections, Plasmodium“[Mesh] OR “Infection, Plasmodium“[Mesh] OR “Plasmodium Infection“[Mesh] OR “Remittent Fever“[Mesh] OR “Fever, Remittent“[Mesh] “Marsh Fever“[Mesh] OR “Fever, Marsh“[Mesh] OR Paludism[Mesh]) | 115 |

Embase 12 June 2023

| No. | Key concept | Search terms | Results |
| --- | --- | --- | --- |
| 1. | Reduced glutathione | ‘reduced glutathione’:ti,ab,kw,de OR  GSH:ti,ab,kw,de OR ‘reduced glutathione’/exp OR GSH/exp OR “gamma-L-Glutamyl-L-Cysteinylglycine”:ti,ab,kw,de OR “gamma L Glutamyl L Cysteinylglycine”:ti,ab,kw,de OR “gamma-L-Glu-L-Cys-Gly”:ti,ab,kw,de OR “gamma L Glu L Cys Gly”:ti,ab,kw,de | 145,840 |
| 2. | Malaria | malaria:ti,ab,kw,de OR plasmodium:ti,ab,kw,de OR ‘Remittent Fever’:ti,ab,kw,de OR ‘Marsh Fever’:ti,ab,kw,de OR Paludism:ti,ab,kw,de OR malaria/exp | 156,216 |
| 3. | 1 AND 2 | (‘reduced glutathione’:ti,ab,kw,de OR  GSH:ti,ab,kw,de OR ‘reduced glutathione’/exp OR GSH/exp OR “gamma-L-Glutamyl-L-Cysteinylglycine”:ti,ab,kw,de OR “gamma L Glutamyl L Cysteinylglycine”:ti,ab,kw,de OR “gamma-L-Glu-L-Cys-Gly”:ti,ab,kw,de OR “gamma L Glu L Cys Gly”:ti,ab,kw,de) AND (malaria:ti,ab,kw,de OR plasmodium:ti,ab,kw,de OR ‘Remittent Fever’:ti,ab,kw,de OR ‘Marsh Fever’:ti,ab,kw,de OR Paludism:ti,ab,kw,de OR malaria/exp) | 649 |

Scopus 13 June 2023

| No. | Key concept | Search terms | Results |
| --- | --- | --- | --- |
| 1. | Reduced glutathione | TITLE-ABS-KEY ( "reduced glutathione" OR "reduced glutathione" OR gsh OR "gamma-l-glutamyl-l-cysteinylglycine" OR "gamma l glutamyl l cysteinylglycine" OR "gamma-l-glu-l-cys-gly" OR "gamma l glu l cys gly" ) | 86,966 |
| 2. | Malaria | TITLE-ABS-KEY ( malaria OR plasmodium OR "plasmodium infection" OR "remittent fever" OR "marsh fever" OR paludism ) | 156,935 |
| 3. | 1 AND 2 | ( TITLE-ABS-KEY ( "reduced glutathione" OR "reduced glutathione" OR gsh OR "gamma-L-Glutamyl-L-Cysteinylglycine" OR "gamma L Glutamyl L Cysteinylglycine" OR "gamma-L-Glu-L-Cys-Gly" OR "gamma L Glu L Cys Gly" ) ) AND ( TITLE-ABS-KEY ( malaria OR plasmodium OR "plasmodium infection" OR "remittent fever" OR "marsh fever" OR paludism ) ) | 285 |

MEDLINE 13 June 2023

| No. | Key concept | Search terms | Results |
| --- | --- | --- | --- |
| 1. | Reduced glutathione AND Malaria | (“reduced glutathione” OR “reduced glutathione” OR GSH OR “gamma-L-Glutamyl-L-Cysteinylglycine” OR “gamma L Glutamyl L Cysteinylglycine” OR “gamma-L-Glu-L-Cys-Gly” OR “gamma L Glu L Cys Gly”) AND (malaria OR plasmodium OR “Remittent Fever“ OR “Marsh Fever“ OR Paludism) | 295 |

Ovid 13 June 2023

| No. | Key concept | Search terms | Results |
| --- | --- | --- | --- |
| 1. | Reduced glutathione AND Malaria | (“reduced glutathione” OR “reduced glutathione” OR GSH OR “gamma-L-Glutamyl-L-Cysteinylglycine” OR “gamma L Glutamyl L Cysteinylglycine” OR “gamma-L-Glu-L-Cys-Gly” OR “gamma L Glu L Cys Gly”) AND (malaria OR plasmodium OR “Remittent Fever“ OR “Marsh Fever“ OR Paludism) {Including Limited Related Terms} | 226 |

ProQuest 13 June 2023

| No. | Key concept | Search terms | Results |
| --- | --- | --- | --- |
| 1. | Reduced glutathione AND Malaria | (“reduced glutathione” OR “reduced glutathione” OR GSH OR “gamma-L-Glutamyl-L-Cysteinylglycine” OR “gamma L Glutamyl L Cysteinylglycine” OR “gamma-L-Glu-L-Cys-Gly” OR “gamma L Glu L Cys Gly”) AND (malaria OR plasmodium OR “Remittent Fever“ OR “Marsh Fever“ OR Paludism) |  |
