# Supplementary material for: Association of reduced glutathione levels with Plasmodium falciparum and Plasmodium vivax malaria: a systematic review and meta-analysis
Source: Sci Rep. 2023 Sep 30;13:16483. doi: 10.1038/s41598-023-43583-z (PMC10542361; doi:10.1038/s41598-023-43583-z)
Supplement: Supplementary file 4 — Supplementary Table S4. [file 41598_2023_43583_MOESM4_ESM.docx]

**Association of reduced glutathione levels with *Plasmodium falciparum* and *Plasmodium vivax* malaria: A systematic review and meta-analysis**

Manas Kotepui^1*^, Kwuntida Kotepui^1^, Aongart Mahittikorn^2*^, Hideyuki J Majima^1^, Jitbanjong Tangpong^1^, Hsiu-Chuan Yen^3^

^1^Medical Technology, School of Allied Health Sciences, Walailak University, Tha Sala, Nakhon Si Thammarat, Thailand

^2^Department of Protozoology, Faculty of Tropical Medicine, Mahidol University, Bangkok, Thailand

^3^ Department of Medical Biotechnology and Laboratory Science, College of Medicine, Chang Gung University, Taoyuan, Taiwan

^4^ Department of Nephrology, Chang Gung Memorial Hospital at Linkou, Taoyuan, Taiwan

***Corresponding author**

Manas Kotepui [manas.ko@wu.ac.th](mailto:manas.ko@wu.ac.th), Tel.: +66954392469

Kwuntida Uthaisar Kotepui: [kwuntida.ut@wu.ac.th](mailto:kwuntida.ut@wu.ac.th)

Aongart Mahittikorn: aongart.mah@mahidol.ac.th

Hideyuki J Majima: [k0941761@kadai.jp](mailto:k0941761@kadai.jp)

Jitbanjong Tangpong: rjitbanj@wu.ac.th

Hsiu-Chuan Yen: yen@mail.cgu.edu.tw

**Table S4. Meta-regression results**

| **Meta-analysis of GSH** | **Covariates** | ***P* value** | **tau2** | **I^2^ (%)** | **R-squared (%)** | **Number of studies** |
| --- | --- | --- | --- | --- | --- | --- |
| **Malaria patients vs uninfected individuals** | Publication years | 0.053 | 4.601 | 99.15 | 0.00 | 17 |
|  | Study designs | 0.968 | 4.042 | 98.96 | 8.19 | 17 |
|  | Continents | 0.794 | 4.246 | 99.04 | 3.56 | 17 |
|  | Participant’s groups | 0.878 | 4.949 | 98.95 | 0.00 | 17 |
|  | *Plasmodium* species | 0.038 | 4.864 | 99.21 | 0.00 | 17 |
|  | Diagnostic method for malaria | 0.569 | 4.965 | 99.27 | 0.00 | 17 |
|  | Quality rank of included studies | 0.664 | 3.395 | 98.79 | 22.88 | 17 |

N/A, not assessed because of collinearity
