# Supplementary material for: Association of reduced glutathione levels with Plasmodium falciparum and Plasmodium vivax malaria: a systematic review and meta-analysis
Source: Sci Rep. 2023 Sep 30;13:16483. doi: 10.1038/s41598-023-43583-z (PMC10542361; doi:10.1038/s41598-023-43583-z)
Supplement: Supplementary file 5 — Supplementary Figure 1. [file 41598_2023_43583_MOESM5_ESM.pdf]

Reduced glutathione levels in relation to *Plasmodium* infection: A systematic review and meta-analysis

Manas Kotepui[1*], Kwuntida Kotepui[1], Aongart Mahittikorn[2*], Hideyuki J Majima[1], Jitbanjong Tangpong[1], Hsiu-Chuan Yen[3]

[1]Medical Technology, School of Allied Health Sciences, Walailak University, Tha Sala, Nakhon Si Thammarat, Thailand

[2]Department of Protozoology, Faculty of Tropical Medicine, Mahidol University, Bangkok, Thailand

[3] Department of Medical Biotechnology and Laboratory Science, College of Medicine, Chang Gung University, Taoyuan, Taiwan

[4] Department of Nephrology, Chang Gung Memorial Hospital at Linkou, Taoyuan, Taiwan

***Corresponding author**

Manas Kotepui manas.ko@wu.ac.th, Tel+ :.66954392469

Kwuntida Uthaisar Kotepui: kwuntida.ut@wu.ac.th

Aongart Mahittikorn: aongart.mah@mahidol.ac.th

Hideyuki J Majima: k0941761@kadai.jp

Jitbanjong Tangpong: rjitbanj@wu.ac.th

Hsiu-Chuan Yen: yen@mail.cgu.edu.tw

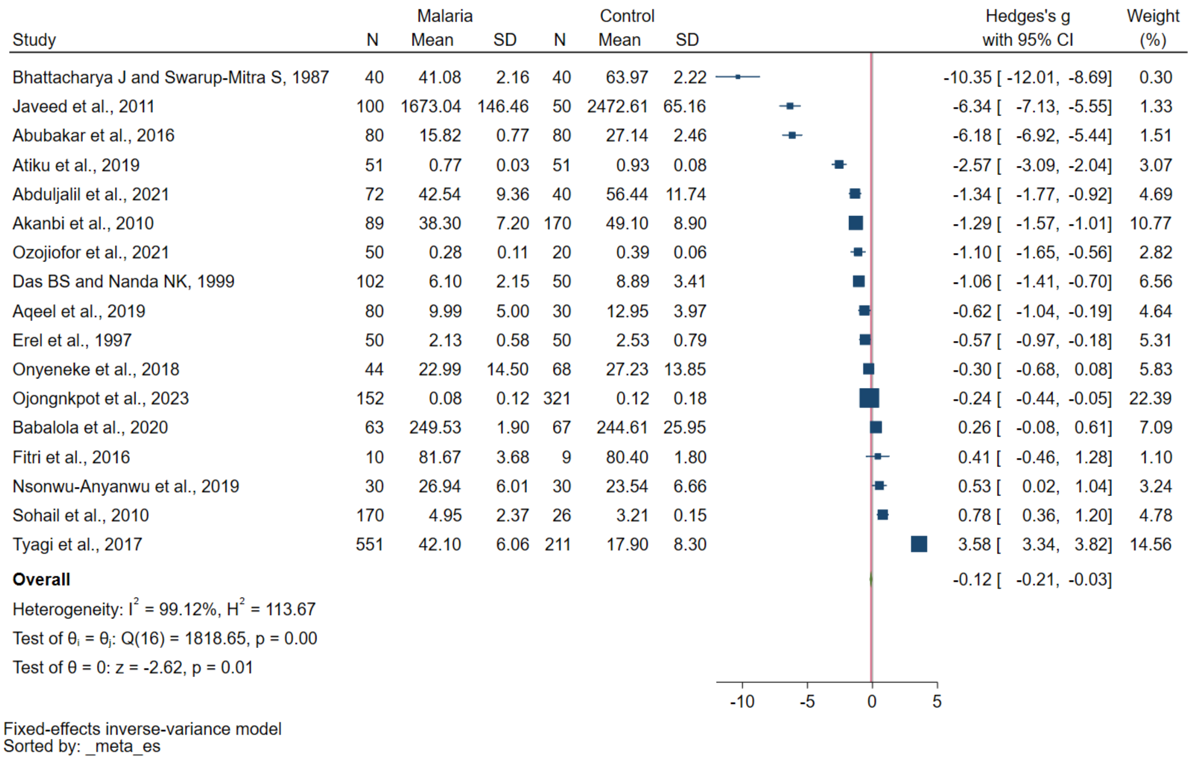

Supplementary Figure 1. The forest plot shows the difference in the reduced glutathione levels between malaria patients and uninfected controls using fixed-effects model. Abbreviation: CI, confidence interval; N, number of participants; SD, standard deviation.
